# Impact of CPAP Therapy on Cognition and Fatigue in Patients with Moderate to Severe Sleep Apnea: A Longitudinal Observational Study

**DOI:** 10.3390/clockssleep6040051

**Published:** 2024-12-23

**Authors:** Oumaïma Benkirane, Olivier Mairesse, Philippe Peigneux

**Affiliations:** 1 UR2NF—Neuropsychology and Functional Neuroimaging Research Unit, at CRCN—Centre for Research in Cognition and Neurosciences and UNI—ULB Neuroscience Institute, Université Libre de Bruxelles (ULB), 1050 Brussels, Belgium; oumaima.benkirane@ulb.be; 2 BBCO-Brain, Body and Cognition, Department of Psychology, Faculty of Psychology and Educational Sciences, Vrije Universiteit Brussel, 1050 Brussels, Belgium; olivier.mairesse@vub.be

**Keywords:** obstructive sleep apnea (OSA), CPAP therapy, cognition, cognitive Fatigue (CF)

## Abstract

Continued solicitation of cognitive resources eventually leads to cognitive fatigue (CF), i.e., a decrease in cognitive efficiency that develops during sustained cognitive demands in conditions of constrained processing time, independently of sleepiness. The expression of CF and its impact on cognition are partly contingent upon prior sleep quality and its restorative effects. Sleep in obstructive sleep apnea (OSA) may be largely restored through the use of continuous positive airway pressure (CPAP) treatment, contributing to a gradual improvement in sleep quality. In this longitudinal observational study, we investigated immediate and longer-term behavioral effects of CPAP treatment on cognitive functioning, evaluating outcomes after the initiation of treatment, and at three and six months, in compliant CPAP-treated OSA patients. Results indicate that CPAP therapy significantly enhances subjective sleep quality and cognitive functions, including episodic memory, inhibition, sustained attention, working memory, and executive control. Noticeable performance improvements were observed in CF-inducing tasks, particularly after six months of CPAP use. Participants also reported substantial gains in quality of life, reduced daytime sleepiness, and improved mood. These results confirm that CPAP therapy not only alleviates immediate physiological disturbances associated with OSA, but also supports cognitive recovery and enhanced overall daily functioning.

## 1. Introduction

Obstructive sleep apnea (OSA) is a condition in which sleep restorative effects are impaired. OSA is a highly prevalent (9–24% of adults) [1] sleep-related breathing disorder (SRBD) defined by recurrent episodes of airflow obstructions ultimately leading to brief arousals, intermittent hypoxemia, snoring, and sleep fragmentation (SF) [2]. Fragmented sleep and nocturnal hypoxemia result in excessive daytime sleepiness and cognitive deficits, mostly prevalent in tasks that require a sustained use of cognitive resources, e.g., sustained attention and executive functions, but SRBD can also exert a deleterious impact on declarative memory, productivity, anxiety, depression, and social interactions [3,4,5,6,7].

One of the primary mechanisms linking OSA to cognitive impairment is the recurrent hypoxia experienced during apneic episodes. Studies indicate that intermittent hypoxia can lead to brain structural and cerebrovascular damage, which may manifest as cognitive deficits. For instance, Karapin and collaborators suggested that recurrent apneic pauses with subsequent intermittent brain hypoxia and sleep fragmentation could affect cognitive impairment, potentially causing brain structural and cerebrovascular damage [8]. Similarly, Wang and collaborators highlighted that hypoxia and sleep disturbances are associated with alterations in gray matter structure, which can further exacerbate cognitive dysfunction in OSA patients [9]. This is supported by findings from Gu and collaborators, who identified specific electroencephalographic biomarkers that correlate with cognitive deficits in individuals with OSA, emphasizing the role of disrupted sleep architecture in cognitive decline [10]. Moreover, reduced slow-wave sleep, a common consequence of OSA, may further impair memory consolidation and exacerbate fatigue, highlighting the complex interplay between disrupted sleep architecture and cognitive dysfunction [11]. OSA has also been linked to circadian rhythm disruptions, which may play a role in the cognitive and fatigue-related consequences of the disorder. A study investigating the key circadian proteins NPAS2 and Rev-Erb-α revealed lower NPAS2 levels in severe OSA patients compared to controls, particularly during REM sleep apnea episodes. This reduction likely disrupts circadian regulation, compounding cognitive deficits and fatigue [12].

Moreover, the impact of OSA on neurocognitive functions is not limited to hypoxia alone. Sleep fragmentation, characterized by frequent arousals during the night, significantly disrupts sleep quality, leading to excessive daytime sleepiness and fatigue. OSA is associated with marked daytime sleepiness and cognitive dysfunction, which can severely affect quality of life [13]. The interaction between sleep fragmentation and metabolic disturbances, such as impaired glucose metabolism, highlights the multifactorial nature of the impact of OSA on cognition and fatigue [14]. Additionally, the reduction in slow-wave sleep observed in OSA may worsen these metabolic disturbances, contributing to daytime fatigue and impaired cognitive performance [11]. The cumulative effect of poor sleep quality and cognitive impairment is further evidenced by a meta-analysis reporting a 26% increased risk of neurocognitive decline in individuals with sleep-disordered breathing [15]. This relationship underscores the importance of sleep quality in maintaining cognitive health.

Symptomatic treatments of OSA such as continuous positive airway pressure (CPAP [16]) can potentially limit its negative consequences and optimize quality of life [5]. Although CPAP treatment leads to a restoration of sleep functions (and limits SF), its impact on cognitive impairment remains poorly investigated, supporting the consensus that it markedly improves SRBD-related daytime sleepiness [17]. Only vigilance was consistently shown to be improved after CPAP use [16]. However, other domains could and should benefit from improved sleep quality after CPAP use, e.g., declarative memory [7]. Notably, mental or cognitive fatigue (CF) is defined as a decrease in cognitive efficiency developing during sustained cognitive demands in conditions of constrained processing time, independently of sleepiness [18]. CF may arise from prolonged duration and/or limited processing time in mentally challenging tasks, potentially leading to subjective fatigue, feelings of “tiredness and lack of energy”, and a decline in performance [19]. Dynamic models of stress and performance [20,21] suggest that drops in performance under high cognitive load conditions are actually associated with disruptions in the access to the pool of brain resources, rather than a mere reduction in these resources. They also suggest that the manifestation of CF is partially dependent on previous sleep conditions and their restorative effects on cognitive functions [22,23].

Up to now, CF has been mostly explored in clinical conditions characterized by chronic fatigue symptoms, such as multiple sclerosis (MS) [24]. Polysomnography studies have established a significant link between fatigue and sleep disorders in MS, and particularly SRBD [25]. The specific links between CF and (OSA-related) sleep disruptions remain quite unexplored, although OSA is a common disorder associated with cognitive deficits, and is thus potentially mediated by CF. In this respect, OSA constitutes a sensible clinical model to assess the relationships between subjective and objective markers of fatigue, and their relationship with cognitive performance in the context of reversible sleep disruptions. To the best of our knowledge, few studies have investigated long-term cognitive effects of CPAP treatment, and none investigated its impact on CF. To address this gap, this longitudinal observational study investigated the effects of CPAP therapy on cognitive fatigue and cognitive performance in patients with moderate to severe OSA. Specifically, we aimed to determine whether CPAP therapy leads to significant improvements in subjective and objective measures of cognitive functioning and fatigue, with evaluations conducted at baseline, 3 months, and 6 months after treatment initiation. We hypothesized that CPAP therapy would lead to improved cognitive performance and reduced cognitive fatigue, with greater effects observed after 6 months of compliant treatment.

## 2. Results

### 2.1. Questionnaire Pre-Testing

Sleep Quality: The Pittsburgh Sleep Quality Index (PSQI) revealed a significant improvement in sleep quality (F(2, 31.10) = 8.49, *p* = 0.001) from Session 1 to Sessions 2 and 3 (*p*’s < 0.05), while the difference between Session 2 and Session 3 was not significant (*p* > 0.05).

Fatigue: The Brugmann Fatigue Scale (BFS) assessed both mental and physical fatigue (defined as behavioral rest propensity). Mental fatigue showed a notable reduction over time (F(2, 28.5) = 15.14, *p* < 0.001) from Session 1 to Sessions 2 and 3 (*p*’s < 0. 001), with no significant difference between Session 2 and Session 3 (*p* > 0.05). Physical fatigue also significantly decreased (F(2, 28.41) = 6.10, *p* = 0.007) from Session 1 to Sessions 2 and 3 (*p*’s < 0.05), while the difference between Session 2 and Session 3 was not significant (*p* > 0.05). The Fatigue Severity Scale (FSS) indicated a significant reduction in the perceived impact of fatigue on various daily functions (F(2, 31.38) = 6.51, *p* = 0.004) from Session 1 to Session 3 (*p* < 0.05), but not between Sessions 1 and 2 or between Sessions 2 and 3 (*p*’s > 0.05).

Circadian preferences: The Chronotype Questionnaire (CHQ) revealed no significant change in behavioral rhythms and sleep pattern preferences across sessions (F(2, 30.68) = 2.25, *p* = 0.12). However, there was a significant shift in distinctness scores (F(2, 31.21) = 11.48, *p* < 0.001) between Session 1 and Sessions 2 and 3 (*p*’s < 0.05), with no significant difference between Sessions 2 and 3 (*p* > 0.05). This suggests a movement from “moderately marked” to “poorly marked–more flexible” time preferences over the sessions.

Anxiety and Depression: The Hospital Anxiety and Depression Scale (HADRS) showed a notable reduction over time (F(2, 33.35) = 3.78, *p* = 0.03) decreasing from mild depression in Session 1 to absence of depression in Session 2 (*p* < 0.05), while the differences between Sessions 1 and 3, and between Sessions 2 and 3, were not significant (*p*’s > 0.05). Anxiety significantly decreased from moderate to minor levels (F(2, 31.42) = 9.71, *p* < 0.001), with significant differences between Session 1 and Sessions 2 and 3 (*p*’s < 0.05), with no significant differences between Session 2 and 3 (*p* > 0.05). Table 1 summarizes the comprehensive results for each questionnaire across the three sessions.

### 2.2. Questionnaires During the Testing Phases

#### 2.2.1. Questionnaire Assessing Sleep Quality of the Preceding Night—St. Mary’s Sleep Questionnaire

Analysis of subjective sleep measures reported on the night before the first day of testing revealed several significant session-related effects. Sleep quality significantly improved (F(1, 36.72) = 6.72, *p* = 0.01) between Session 1 and Session 3 (*p* < 0.05), with no significant changes observed in other session comparisons (*p*’s > 0.05). Subjective sleep depth also significantly enhanced (F(1, 17.89) = 5.79, *p* = 0.03) from Session 1 to Sessions 2 and 3 (*p*’s < 0.05), though the difference between Sessions 2 and 3 was not significant (*p* > 0.05). Subjective sleep duration improved (F(1, 35.70) = 5.51, *p* = 0.03) between Session 1 and Sessions 2 and 3 (*p*’s < 0.05), with no significant changes observed in other session comparisons (*p*’s > 0.05). Additionally, the number of CPAP intercurrent awakenings significantly decreased (F(1, 21.19) = 4.65, *p* = 0.04) between Sessions 1 and 3, with no significant differences for other session comparisons (*p*’s > 0.05).

**Figure 1:**
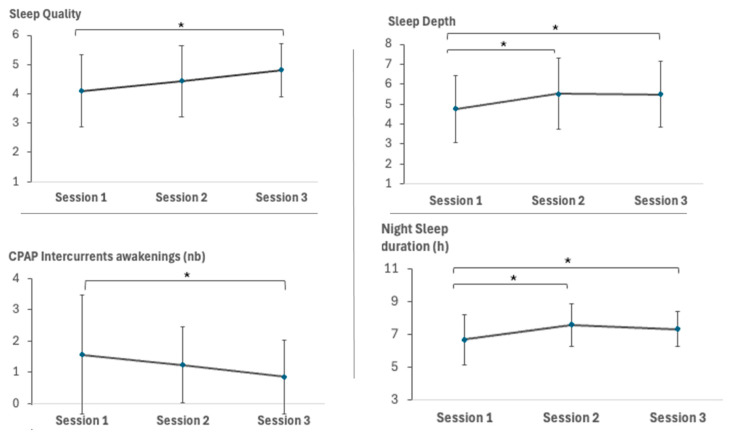
Subjective evaluations from the St-Mary’s Sleep Questionnaire. Evaluations of sleep quality, sleep depth, number of intercurrent awakenings resulting from the CPAP use, and night sleep duration in hours, assessed on the first day of each session. Note: Session 1 = start of the experiment, Session 2 = after three months of CPAP treatment, Session 3 = after six months of CPAP treatment. For Sleep Depth scores, 1 = very light, 2 = light, 3 = quite light, 4 = moderately light, 5 = moderately deep, 6 = quite deep, 7 = deep, 8 = very deep. For Sleepiness scores, 1 = very sleepy, 2 = quite sleepy, 3 = a little sleepy, 4 = lucid, 5 = alert, 6 = very alert. * *p* < 0.05. Error bars represent standard errors.

The results are illustrated in Figure 1. For a detailed breakdown of the St. Mary’s Sleep Questionnaire results for each session, refer to Tables S1 and S2 in the Supplementary Material subsection.

#### 2.2.2. Questionnaire Assessing CPAP Use

CPAP Adaptation and Device Usage: Participants were surveyed about their experiences with CPAP use at three intervals: within the first 15 days, after 3 months, and after 6 months. The majority of participants adapted to CPAP at varying rates, with eleven adapting within a week, three within 2 weeks, another three within a month, two within 2 months, and three taking over 6 months. The devices used varied among participants: seventeen were using the ResMed AirSense 10 AutoSet model, one used the DeVilbiss Blue AutoPlus, one used a Weinmann device, and one used the Löwenstein Prisma Smart. These devices are recognized for their reliability and features such as auto-adjusting pressure and customizable settings, allowing them to adapt to individual patient needs and ensure effective and comfortable therapy. A LMM conducted on the mean usage of CPAP treatment across the three sessions showed no significant differences (F(2, 33.74) = 0.19, *p* > 0.05). Detailed results are presented in the Participants section.

Impact of Diagnosis and CPAP Treatment on Life: The perceived impact of the OSA diagnosis and CPAP treatment on participants’ daily lives was assessed using a scale where 1 = Dramatic improvement; 2 = Moderate change; 3 = Bad effects; and 4 = Disappointing, this device is too constraining. Over the study period, this impact changed significantly (F(2, 32.43) = 4.12, *p* = 0.03). Initially, at Session 1, participants reported a more negative effect on their quality of life. However, this perception improved significantly after three months of CPAP use, indicating a reduction in negative impact (*p* = 0.05). No significant differences were observed between the other sessions (*p*’s > 0.05), suggesting that the most notable improvement occurred at the initiation of the treatment.

##### CPAP Treatment Outcomes

A generalized linear mixed model (GLMM) with a binomial family and a logit link function was utilized to analyze the binary outcome data. Nine observations were excluded due to missing values. The analysis found that general discomfort from the mask (pain, dryness, discomfort) decreased significantly over time ($\chi^{2}$ = 11.69, *p* < 0.01). Mood improved significantly across sessions ($\chi^{2}$ = 10.02, *p* < 0.01). Sudden bouts of fatigue showed a trend towards reduction, especially after 3 months ($\chi^{2}$ = 5.47, *p* = 0.06). No significant changes were observed for other factors, including practical constraints, inconvenience for the spouse, mask usage during the night, and daytime issues such as sleepiness, concentration, migraines, and shortness of breath (all *p*’s > 0.05). Detailed results from the CPAP use questionnaire are presented in Table 2.

Detailed results of the correlation analysis between CPAP usage and factors of the feedback questionnaire are presented in Table 3.

### 2.3. Neuropsychological Evaluation

**Figure 2:**
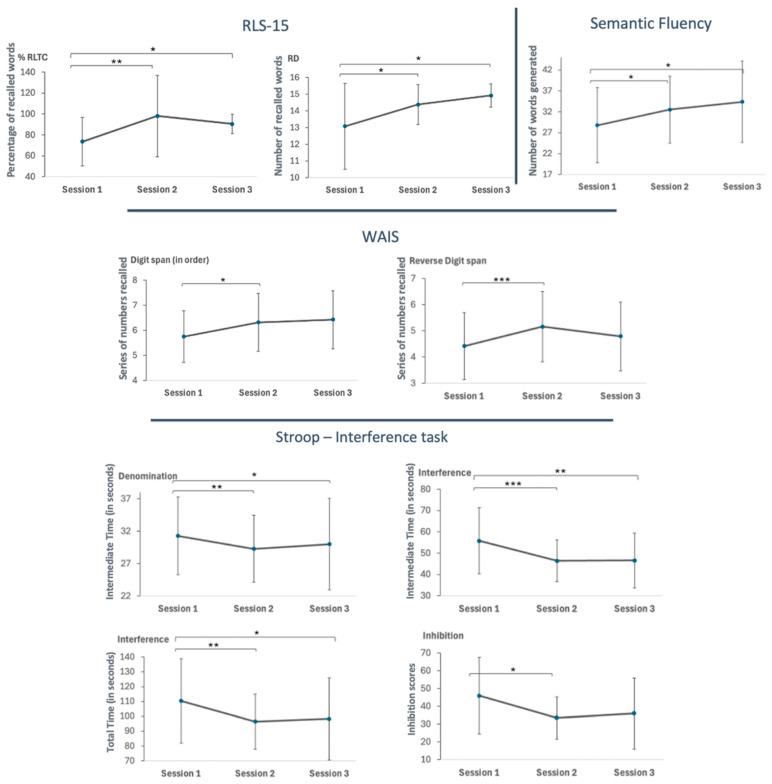
Evolution of neuropsychological test outcomes across sessions. **Top Panel (Left):** RLS-15 task scores showing the percentage of correctly recalled words (RLTC) and the number of correctly classified words (RD) across sessions. **Top Panel (Right):** Number of words generated in the Semantic Fluency task. **Middle Panel (Left and Right):** Digit span (forward and reverse) performance from the WAIS test across sessions. **Bottom Panel:** Stroop task scores, including denomination time, interference (intermediate and total time), and inhibition scores. Note: Session 1 = start of the experiment, Session 2 = after three months of CPAP treatment, Session 3 = after six months of CPAP treatment. * *p* < 0.05, ** *p* < 0.01, *** *p* < 0.001. Error bars represent standard errors.

A LMM was conducted on neuropsychological test outcomes across three sessions. Key results are summarized in Table 4 and illustrated in Figure 2.

Episodic Memory: Analysis of the RLS-15 test showed a significant difference in the percentage of words recalled consecutively in immediate recall trials (% RLTC), with significant improvement from Session 1 to Sessions 2 and 3 (*p*’s < 0.05), and no further significant changes between Sessions 2 and 3 (*p* > 0.05). Delayed recall (RD) also displayed a significant difference over time (F(2, 20) = 3.70, *p* = 0.04,), improving from Session 1 to Sessions 2 and 3 (*p*’s < 0.05), with no further significant changes between Sessions 2 and 3 (*p* > 0.05). The average words correctly recalled during immediate recall trials (RM) did not vary significantly (*p* > 0.05).

Inhibition: For the intermediate time for denomination of the Stroop task (DenoTI), there was a significant decrease (F(2,32.33) = 6.51, *p* = 0.004) from Session 1 to Sessions 2 and 3 (*p*’s < 0.05), with no significant differences between Sessions 2 and 3 (*p* > 0.05). Other scores related to the denomination subtask did not differ across sessions (see Table S3–Supplementary Material).

In the interference subtask, scores showed a significant reduction in intermediate time (InterTI) (F(2,33.68) = 13.97, *p* < 0.001) from Session 1 to both Sessions 2 and 3 (*p*’s < 0.05), with no significant difference between Sessions 2 and 3 (*p* > 0.05). For total time (InterTT), interference scores also decreased significantly (F(2,34.63) = 6.55, *p* = 0.004) from Session 1 to Sessions 2 and 3, but there was no significant difference between Sessions 2 and 3 (*p* > 0.05).

In terms of inhibition, measured as the difference in response speed between denomination and interference conditions, scores showed a significant reduction in time (F(2,35.83) = 3.25, *p* = 0.05) from Session 1 to Session 2 (*p* < 0.05); comparisons between the other sessions did not differ (*p*’s > 0.05).

Short-Term and Working Memory: For the digit span in order, a significant effect was observed (F(2, 35.85) = 3.35, *p* = 0.05), with scores increasing from Session 1 to Session 2 and 3) (*p* < 0.05), with no significant differences between other sessions (*p*’s > 0.05). The reverse digit span showed a significant session effect (F(2, 36.27) = 7.92, *p* = 0.002), with results increasing from Session 1 to Session 2 (*p* < 0.01); comparisons between the other sessions did not differ (*p*’s > 0.05).

Verbal Fluency: Semantic fluency displayed a significant increase (F(2, 36.23) = 7.41, *p* = 0.002) between Session 1 and both Sessions 2 and 3 (*p*’s < 0.05), while the difference between Sessions 2 and 3 was not significant (*p* > 0.05). Phonological fluency remained stable across sessions (*p* > 0.05).

Vigilance: The Psychomotor Vigilance Task (PVT) scores did not show significant changes (see Table S4, Supplementary Material).

### 2.4. TloadDback Task

#### 2.4.1. Pre-Test Estimating the Interstimulus Interval (ISI)

The interstimulus interval (ISI) was measured on the first morning of each of the three sessions (1 × 3 times) to assess the participant’s cognitive load limit, defined as the time needed to obtain a score of less than 85% across three consecutive blocks. This measurement allowed for the appropriate adjustment of the cognitively fatiguing task for the following day.

The LMM of the ISI results, expressed in seconds, revealed significant differences between sessions (F(2, 35.88) = 16.27, *p* < 0.001), with the ISI significantly decreasing between Sessions 1 and 2 (*p* < 0.05), between Sessions 1 and 3 (*p* < 0.001), and between Sessions 2 and 3 (*p* < 0.001).

#### 2.4.2. Performance in the TloadDback Task

On day two of each session, participants performed a cognitive fatigue-inducing task tailored to their individual ISI. During these sessions, data were collected over 16 min of the TloadDback task. These data were segmented into four 4 min intervals to evaluate how fatigue markers evolved over time as participants performed the task.

**Figure 3:**
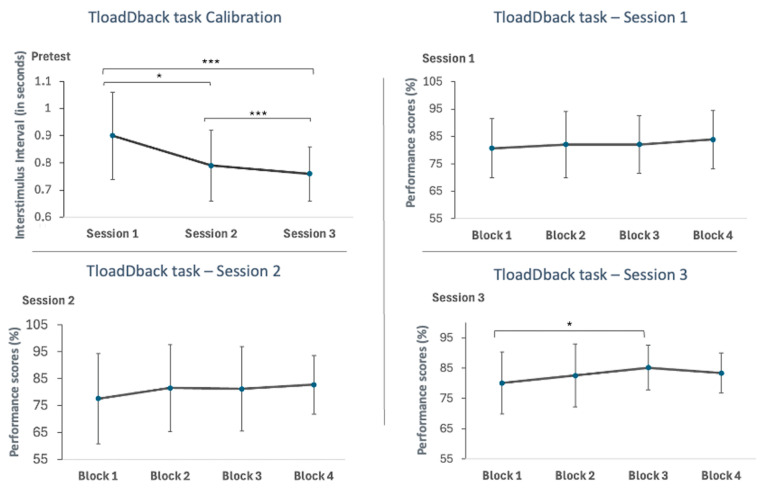
**Top Left Panel:** Interstimulus interval in seconds in the calibration task of the TloadDback task across sessions. **Top Right Panel:** Performance in percent during the four blocks of the TloadDback task during Session 1. **Bottom Left Panel:** Performance in percent during the four blocks of the TloadDback task during Session 2. **Bottom Right Panel:** Performance in percent during the four blocks of the TloadDback task during Session 3. Note: Session 1 = start of the experiment, Session 2 = after three months of CPAP treatment, Session 3 = after six months of CPAP treatment. * *p* < 0.05, *** p < 0.001. Error bars represent standard errors.

An LMM was conducted to examine performance across these different blocks, revealing a significant difference only within the blocks of Session 3 (F(3, 45) = 2.80, *p* = 0.05). Post hoc comparisons using the Holm–-Bonferroni test indicated a significant improvement in performance from the first 4 min block to the third 4 min block (*p* < 0.05). However, no significant differences were found in the following comparisons: block 1 vs. block 2, block 1 vs. block 4, block 2 vs. block 3, block 2 vs. block 4, and block 3 vs. block 4 (all *p*’s > 0.05). Detailed results of the interstimulus interval and performance across blocks in Session 3 are illustrated in Figure 3.

We also computed the Deltas of performance by subtracting the performance in the last block from that in the first block for each of the three sessions. The LMM conducted on these Delta scores revealed no significant differences across sessions (F(2, 37.36) = 0.37, *p* = 0.69).

#### 2.4.3. Relationship Between CPAP Adherence and Cognitive Outcomes

To further explore the relationship between CPAP adherence and cognitive performance, we conducted correlation analyses focusing on average CPAP use and cognitive performance indicators (Table 5).

#### 2.4.4. Subjective Evaluations of Sleepiness, Fatigue, Stress, and Motivation Following the TloadDback Task

Paired samples *t*-tests on the subjective evaluations of sleepiness before and after the TloadDback task revealed a significant increase in sleepiness for Session 1 (t(23) = −3.76, *p* = 0.001) and Session 2 (t(20) = −3.22, *p* = 0.004). However, there was no significant change in sleepiness for Session 3 (t(14) = −1.06, *p* = 0.31). Additionally, we computed the Delta for sleepiness by subtracting pre-task sleepiness from post-task sleepiness for each session. The LMM of these Delta scores showed no significant differences across sessions (F(2, 35.77) = 2.08, *p* = 0.14).

Paired samples *t*-tests on the subjective evaluation of cognitive fatigue before and after the TloadDback task revealed significant increases in cognitive fatigue for Session 1 (t(23) = −3.55, *p* = 0.002), Session 2 (t(20) = −2.47, *p* = 0.02), and Session 3 (t(14) = −2.23, *p* = 0.04). Additionally, we computed the Delta for cognitive fatigue by subtracting pre-task cognitive fatigue from post-task cognitive fatigue for each session. The LMM of these Delta scores showed no significant differences across the sessions (F(2, 37.10) = 0.03, *p* = 0.97).

Paired samples *t*-tests on the subjective evaluation of stress before and after the TloadDback task showed a significant increase in stress for Session 1 (t(23) = −5.42, *p* < 0.001), Session 2 (t(20) = −3.55, *p* = 0.002), and Session 3 (t(14) = −3.65, *p* = 0.003). Additionally, we computed the Delta for stress by subtracting pre-task stress from post-task stress for each session. The LMM of these Delta scores revealed no significant differences across sessions (F(2, 36.61) = 0.82, *p* = 0.45).

**Figure 4:**
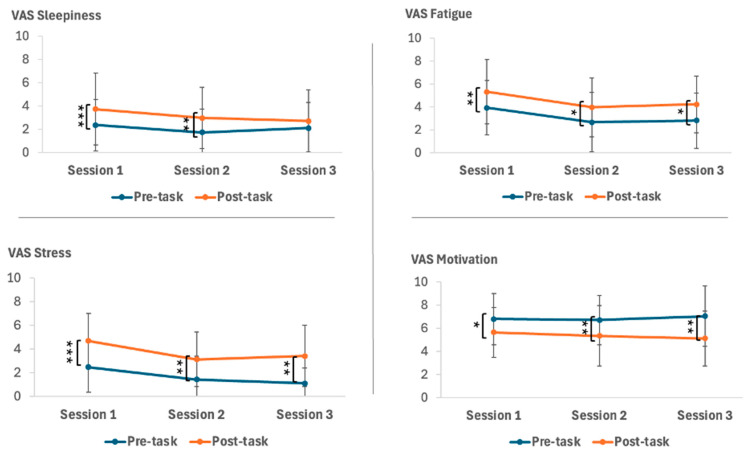
Visual analog scales before and after the TloadDback task. **Top Left Panel:** Subjective evaluation of the sleepiness pre- and post-task. **Top Right Panel:** Subjective evaluation of the cognitive fatigue pre- and post-task. **Bottom Left Panel:** Subjective evaluation of the stress pre- and post-task. **Bottom Right Panel:** Subjective evaluation of the motivation pre- and post-task. Note: Session 1 = start of the experiment, Session 2 = after three months of CPAP treatment, Session 3 = after six months of CPAP treatment. * *p* < 0.05, ** p < 0.01, *** *p* < 0.001. Error bars represent standard errors.

Paired samples *t*-tests on the subjective evaluation of motivation before and after the TloadDback task revealed a significant decrease in motivation for Session 1 (t(23) = 2.23, *p* = 0.03), Session 2 (t(20) = 2.77, *p* = 0.01), and Session 3 (t(14) = 2.92, *p* = 0.01). Additionally, we computed the Delta for motivation by subtracting pre-task motivation from post-task motivation for each session. The LMM of these Delta scores showed no significant differences across sessions (F(2, 55) = 069, *p* = 0.51). The comprehensive results of the paired samples *t*-tests are summarized in Table 6 and Figure 4.

## 3. Materials and Methods

This longitudinal observational study aimed to evaluate the effects of CPAP therapy on cognitive fatigue and cognitive performance in patients with moderate to severe obstructive sleep apnea. Participants were assessed at three key time points: baseline (within the first two weeks following CPAP initiation), and at 3 months and 6 months of CPAP treatment. This study focused on both subjective and objective measures of cognitive functioning and fatigue, using standardized neuropsychological tests, cognitive fatigue induction protocols, and self-reported questionnaires.

### 3.1. Study Setting

This study was conducted between 29 April 2021 and 31 May 2023. Participants were recruited through two main channels:

1. Posters and flyers displayed in sleep laboratories in Brussels, targeting individuals spending the night for polysomnography testing.
2. Social media campaigns aimed at individuals recently diagnosed with moderate to severe OSA.

### 3.2. Participants

Inclusion criteria were a recent PSG-based OSA diagnosis with an obstructive apnea-hypopnea index (OAHI) > 15/hour during sleep, and being under CPAP treatment for less than a month. PSG assessments were conducted in certified sleep laboratories and followed the American Academy of Sleep Medicine (AASM [26]) guidelines. Evaluated sleep parameters evaluated included brain activity (electroencephalogram), eye movements (electrooculogram), muscle tone (electromyogram), heart activity (electrocardiogram), airflow, respiratory effort, oxygen levels in the blood, and body position during sleep. Scoring was performed according to the AASM [26] manual, ensuring accurate classification of apneas and hypopneas. CPAP titration was also conducted in the sleep laboratory to determine the optimal pressure required to prevent apneas, hypopneas, and snoring while ensuring patient comfort. This process adhered to the AASM guidelines and was performed by trained technicians. Depending on the specific protocols of the laboratories, titration was carried out either manually or using automatic CPAP devices. Note that PSG assessment and CPAP prescription were independently done at the clinical phase, before we recruited the participants in our study.

Exclusion criteria were prior PAP treatment, neurological or psychiatric conditions, neurodegenerative dementia syndrome, opioid treatment history, or current benzodiazepine intake. Participants were asked to avoid alcohol the day before and during the experimental days. Participants received EUR 40 compensation for their participation.

A total of 45 individuals were initially screened for eligibility based on their OSA diagnosis and CPAP initiation. Thirty met the inclusion criteria, and 22 French-speaking participants (12 females, 10 males; mean age: 46.64 ± 10.66 years, range: 25–65 years; mean OAHI: 39.92 ± 23.94, range: 20–130) were included in this study. Participants had been under CPAP treatment for less than 15 days at baseline, with an average compliance of 6.26 ± 1.67 h per night. Out of these 22 participants, 21 (95.45%) completed the three-month follow-up session (T2; mean age: 46.62 ± 10.93 years, range: 25–65 years; mean OAHI: 39.49 ± 24.44, range: 20–130; compliance: 6.41 ± 1.82 h per night). One participant could not participate in T2 due to frequent work-related travel. Sixteen participants (72.73%) completed the six-month follow-up session (T3; mean age: 48.56 ± 11.1 years, range: 25–65 years; mean OAHI: 42.77 ± 26.47, range: 22–130; compliance: 6.08 ± 1.26 h per night). Reasons for attrition between T2 and T3 included:

- One participant discontinued participation, finding the study too demanding.
- One participant stopped CPAP treatment after significant weight loss and lifestyle improvements.
- Four participants were unavailable due to personal and professional commitments.

### 3.3. Experimental Procedure

#### 3.3.1. Pre-Testing Questionnaires

As the experimental tasks were verbal, participants’ French fluency was informally assessed by the main experimenter (OB) during the initial interview when explaining the modalities of the experiment. At each time point ((1) start of the experiment, (2) after three months of CPAP treatment, and (3) after six months of CPAP treatment), participants completed a battery of psychometric instruments, including the Chronotype Questionnaire (CHQ, circadian typology and individual preferences for morning or evening activities; [27]), the Pittsburgh Sleep Quality Index (PSQI, sleep quality of the previous month, including aspects such as sleep latency, duration, disturbances, and overall satisfaction [28], the Fatigue Severity Scale (FSS, perceived impact of fatigue on daily functioning over the previous month [29]), the Brugmann Fatigue Scale (BFS, the behavioral impact of physical and mental fatigue [30], and the Hospital Anxiety and Depression Scale (HADRS, symptoms of anxiety and depression [31]).

#### 3.3.2. Testing Procedure

**Figure 5:**
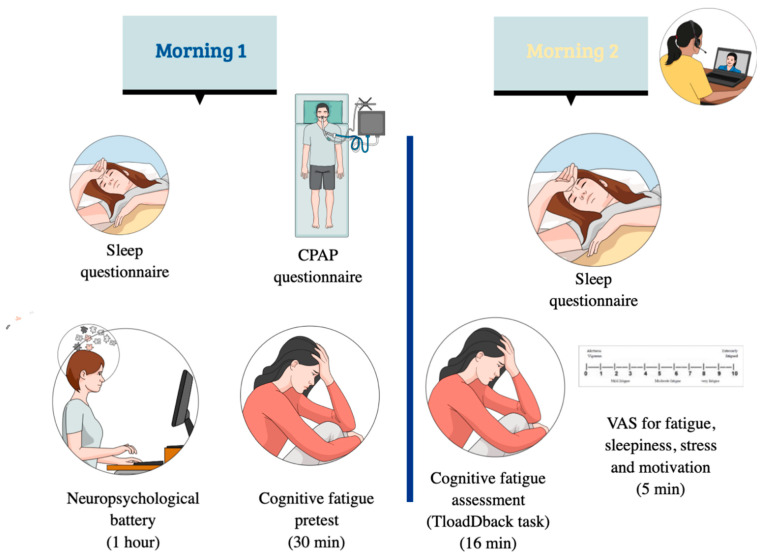
Experimental protocol. Note: VAS = visual analog scales (fatigue, sleepiness, stress, and motivation), each completed two times (before and after the TloadDback task). The same protocol took place after three then six months of CPAP use.

During each of the three experimental sessions (identical procedure), the experiment spanned two successive mornings per participant, to avoid bias linked to the accumulation of fatigue experienced throughout the day and to control for inter-individual circadian variability (Figure 5). To ensure safety during the COVID-19 pandemic, all testing was conducted online using the secure platform at https://meet.jit.si (accessed on 29 April 2021) for communication and Pavlovia [32], for administrating computerized tasks that we coded and made available as open access. A standardized script was used to ensure uniform verbal instructions across participants, while identical written instructions were provided for all questionnaires and online tests. Links to tasks were provided in relevant sections of the text. Video call and screen sharing enabled the main experimenter (OB) to closely monitor the testing process.

On the first morning, patients completed two online questionnaires: an adapted version of the St-Mary’s Sleep Questionnaire [33], to subjectively assess their previous night’s sleep quality, and a questionnaire gathering early feedback of their CPAP use (e.g., the time to adapt, duration of use, difficulties encountered, observed effects), both designed using LimeSurvey [34]. Patients then underwent a neuropsychological assessment battery (see below) and the calibration part of the TloadDback protocol to determine their maximal working memory processing capacity (see below) [18].

The second session took place the following morning. Participants again completed the online version of the St-Mary’s Sleep Questionnaire [33], followed by the TloadDback CF-induction protocol. Cognitive fatigue (CF) was assessed using visual analog scales for fatigue (VASf [35]), sleepiness (VASs), stress (VASst), and motivation (VASm) before and after the task to control for potential confounders. The same procedure was repeated for the sessions held three and six months later.

#### 3.3.3. Experimental Questionnaires

##### Sleep and CPAP Questionnaires

Every morning throughout the entire experimental period, subjects completed the St-Mary’s Sleep Questionnaire [33], comprising 16 questions subjectively assessing the quality of the preceding night. Only on the first morning of each session, participants completed the CPAP feedback questionnaire, comprising 8 questions subjectively assessing the accommodation towards CPAP treatment.

##### Neuropsychological Evaluation

Neuropsychological evaluations covering verbal and visual short-term and working memory, episodic memory, and attentional and executive functions were administered on the first morning of each session. Episodic memory was tested using the RLS-15 [36], where participants memorized a list of 15 words and recalled it until they could reproduce it twice consecutively or after 10 trials (immediate recall). A delayed recall test followed 30 min later. Outcome measures included the average number of words correctly retrieved during immediate recall trials (RM), the percentage of words recalled in at least two consecutive immediate recall trials out of total words recalled (% RLTC), and the number of words correctly recalled during the delayed recall (RD).

During the 30 min interval between immediate and delayed recalls, participants completed several tasks. Verbal short-term memory was evaluated using the digit span subtest of the WAIS [37], where span performance was defined by the longest sequence of numbers repeated twice in the correct order. Working memory performance was studied using the reverse digit span subtest of the WAIS [37], where participants reproduced digits in reverse order, with span performance defined as the longest reverse sequence repeated twice correctly. Vigilance was assessed using the Psychomotor Vigilance Task (PVT [38]), measuring sustained attention and reaction time. Participants stopped a clock appearing at random intervals of 2–10 s for 10 min. Inhibition was evaluated with the Stroop task (French version [39] Godefroy, 2012), which included three conditions: (1) naming colored rectangles, (2) reading color names, and (3) naming the print color of words while ignoring the written word. Outcome measures included completion times and errors. After the delayed recall of the RLS-15, verbal fluency tests [39] were administered. Participants had two minutes to generate as many words as possible, starting with the letter “p” (phonological fluency) or belonging to the category of animals (semantic fluency).

##### Cognitive Fatigue-Inducing TloadDback Task

The TloadDback task combines a conventional N-back working memory updating task with an odd/even number decision task [18]. Merging these tasks requires continuous recruitment of working memory resources, influenced by the pace of information processing. A detailed description of the task can be found in [18] (Study 1); only essential information is presented here.

In the TloadDback task, 30 digits and 30 letters per block alternate on the screen (e.g., N–2–X–7–X–1–L…). Participants must (a) press the space key with their left hand when the current letter matches the previous one (1-back task, e.g., …X–7–X…), and (b) use their right hand to indicate whether the digit is odd or even by pressing “2” or “3” on the numeric keypad. Cognitive resource recruitment is individually tailored during a calibration session by adjusting the item presentation speed (interstimulus interval, ISI) to the fastest rate at which the participant can still perform successfully (i.e., achieve an average accuracy per block > 85%, with 65% weight given to the 1-back component and 35% to the odd/even decision component).

During the calibration session on the first experimental day, participants first practiced the 1-back and odd/even decision tasks separately before combining them. Then, they performed the TloadDback task for up to 20 blocks, starting with an ISI of 1500 ms. If participants achieved ≥85% accuracy for a block, the ISI for the next block was reduced by 100 ms, increasing task difficulty by shortening the processing time allowed. Blocks were administered following this staircase procedure until a score of <85% was obtained over three consecutive blocks, indicating that the participant’s cognitive load limit had been reached. The open-access version of the TloadDBack calibration task is available at [https://run.pavlovia.org/benkiraneo/tload_calibration/html] (accessed on 29 April 2021).

Participants underwent a 16 min session of the TloadDback task on the subsequent day. The ISI for all blocks was set to the last successful ISI from the calibration session, increased by 100 ms. The ISI is uniquely tailored to each participant’s maximum processing capacity. Through individual calibration, we ensured that the task remained challenging for every participant. It is important to note that because the ISI varies among individuals, the number of blocks processed during the 16 min practice session also varies accordingly.

##### TloadDback-Related Visual Analog Scales

As stated above, 10 cm long visual analog scales (VAS) were administered on the second day of the experimental sessions immediately before and after the TloadDback task. At each step, 4 VAS were assessed for fatigue (VASf [35]; from perfectly rested to completely exhausted), sleepiness (VASs; from very alert/vigilant to very sleepy), stress (VASst; from not at all stressed to very stressed), and motivation (VASm; from not at all motivated to highly motivated). To control for inter-individual differences in the subjective conceptions of fatigue and sleepiness, cognitive fatigue was described to the participants as “the necessity to cease persistent cognitive efforts without the urge to fall asleep”, while sleepiness was defined as “an intermediate state between waking and sleeping characterized by a tendency to doze off”. The open-access version of the TloadDBack task and visual analog scales is available at [https://run.pavlovia.org/benkiraneo/tload_test/] (accessed on 29 April 2021).

### 3.4. Statistical Analyses

Two types of statistical analyses were performed to evaluate the data collected from the three sessions, i.e., (1) start of the experiment (T1), (2) after three months of CPAP treatment (T2), and (3) after six months of CPAP treatment (T3). All continuous variables, including age and OAHI, were analyzed in their original form without categorization. Mean values and ranges are reported to provide a clear overview of the data distribution. Comparisons between sessions for continuous data were conducted using a linear mixed model (LMM). This study was primarily exploratory, with the aim of generating hypotheses rather than confirming them. Due to the absence of prior data to estimate effect sizes for cognitive fatigue in the context of sleep apnea, systematic a priori power calculations were not conducted. However, post hoc power analyses indicated that the sample sizes and study design were sufficient to detect medium effect sizes [40] with a power (1—β) of at least 0.80 at an α level of 0.05. For the TloadDback task, which included data from 21 participants across two sessions (e.g., T1 and T2), a mean correlation of 0.66 between measures was observed, enabling detection of effects as small as 8.8% of the explained variance. Similarly, for the 16 participants with complete data across three sessions (T1, T2, and T3), a mean correlation of 0.61 was observed, allowing detection of effects as small as 8.0% of the explained variance.

To account for missing data, assumed to be missing at random, LMMs were fitted with “Session” as a fixed effect and “Subjects” as a random effect grouping factor, incorporating all available data points using the lme4 package in R [41] within the JASP interface. The dataset included 48 data points from 16 participants with three measures and 42 data points from 21 participants with two measures, resulting in a total of 90 data points. This approach allowed the inclusion of all available data points without listwise deletion, ensuring robust analyses despite minimal attrition.

Type III sums of squares were used to test model terms via likelihood ratio tests. Restricted maximum likelihood (REML) estimation was employed to fit the models. Covariance structures were estimated from the data [41]. Model fit was assessed using the Akaike Information Criterion (AIC) and examination of residual plots to ensure normality and homoscedasticity. *p*-values were adjusted using the Holm method to control the familywise error rate, and statistical significance was determined at an α level of 0.05. Post hoc pairwise comparisons between sessions were conducted with *t*-tests, and the Holm method was used to adjust *p*-values for multiple comparisons. For binary data, a generalized linear mixed model (GzLMM) with a binomial family and logit link function was utilized. While no explicit sensitivity analyses were conducted, the use of a linear mixed model (LMM) and generalized linear mixed model (GzLMM) inherently ensured the robustness of the findings by accounting for missing data without requiring listwise deletion.

In addition, correlation analyses were conducted to explore relationships between CPAP adherence, measured as mean usage in hours, and self-reported measures of subjective well-being across the three time points (T1, T2, and T3). All estimates are reported as unadjusted. Potential confounders, such as age, sex, and OSA severity, were not included in the models due to the relative homogeneity of the sample, which consisted exclusively of individuals with moderate to severe OSA. No subgroup or interaction analyses were performed, as the relatively homogeneous nature of the sample limited the need for such analyses.

An exploratory factor analysis (EFA) was conducted to identify the underlying structure of the 17 binary variables from the CPAP feedback questionnaire. The analysis was performed in JASP using minimal residual factoring as the extraction method, promax rotation, and a polychoric/tetrachoric correlation matrix. The EFA yielded four factors with the eigenvalues and corresponding variables as depicted in Table 7.

Two variables (reduced daytime sleepiness at work and decrease in shortness of breath) did not load significantly on any of the factors, and were excluded from the final analysis. These variables might represent unique aspects not captured by the identified factors or may reflect specific issues not addressed by the current factor structure.

Pearson correlation coefficients were calculated to evaluate the strength and direction of associations between the continuous factors derived from the exploratory factor analysis and the continuous variable representing the number of hours of CPAP use.

## 4. Discussion

This study aimed to explore the cognitive and subjective effects of continuous positive airway pressure (CPAP) therapy over a six-month period in individuals with obstructive sleep apnea (OSA). As the gold standard treatment for mild to severe OSA [42,43], CPAP is expected to not only alleviate physical symptoms but also impact cognitive functioning. While the improvements in cognitive functioning were particularly evident after six months of CPAP treatment, positive effects were already observed after three months, consistent with previous results showing that CPAP therapy not only alleviates physical symptoms, but also has positive effects on cognitive function. Additionally, the age range of participants (25–65 years) reflects a typical clinical population of OSA patients, with no extreme age groups (very young or very old) included. This balanced distribution reduces the potential confounding effect of age on cognitive improvements observed with CPAP therapy, as significant age-related variations in treatment efficacy are less likely within this range. While younger participants were less represented, the absence of extreme age groups ensures that age is not a major factor influencing the consistency of cognitive outcomes across participants. Studies have indicated that CPAP treatment can improve cognitive function in patients with mild cognitive impairment and OSA [44]. Here, episodic memory, assessed using the RLS-15 task, demonstrated significant improvements in immediate and delayed recall scores after 3 months of treatment, which were maintained after 6 months of CPAP use. This suggests that extended CPAP use may enhance learning and memory retention, potentially due to improved sleep quality and reduced sleep apnea events [45]. Previous research supports these results, showing that CPAP therapy positively impacts cognitive functions by improving sleep architecture [45]. Notably, studies have shown significant improvements in working memory, long-term verbal memory, and short-term visuospatial memory after three months of CPAP therapy [46]. Furthermore, longer durations of CPAP use (exceeding six hours) have been associated with reduced sleepiness, improved daily functioning, and normalization of memory [47]. Our findings demonstrate that CPAP therapy leads to significant improvements in cognitive fatigue and performance, aligning with our objective of assessing the longitudinal impact of CPAP therapy on both subjective and objective measures of cognitive functioning and fatigue in patients with moderate to severe OSA.

Despite improvements in some cognitive domains, the lack of significant changes in working memory after six months suggests that these effects may plateau, as observed in both the reverse digit span subtest of the WAIS and the digit span in order. Significant improvements in reverse span scores were observed after three months of CPAP use, indicating enhanced working memory capabilities over the treatment period, consistent with the idea that improved sleep quality can enhance executive functions, including working memory [48,49]. However, these improvements were no longer significant after six months of CPAP treatment, which is consistent with previous studies showing mixed results regarding the effects of CPAP therapy on working memory, short-term memory, and overall cognitive functioning [50].

The Stroop task demonstrated significant improvements in inhibition after three months of treatment, indicating enhanced cognitive interference management over time with CPAP treatment. This may be attributed to improved sleep quality and cognitive functioning resulting from the alleviation of OSA symptoms. These findings are consistent with research indicating that CPAP therapy can improve cognitive functions, including executive functions and memory [48,49]. These improvements are also consistent with previous findings linking sleep deprivation to top-down alterations in cognitive processes [51].

The results from the Psychomotor Vigilance Task (PVT) following CPAP treatment showed no significant improvement after 3 or 6 months of CPAP use. While the PVT has demonstrated sensitivity to factors like CPAP treatment and vigilance enhancement over time, previous studies have suggested that the PVT may not be sufficiently sensitive to moderate sleep disturbances [49]. In our study, the lack of significant improvement in the PVT task performance may reflect a ceiling in effect performance in participants who presented relatively preserved vigilance at baseline. This limitation suggests that their initial performance levels left limited room for observable improvement. Moreover, the persistent influence of residual sleep fragmentation, variability in individual responsiveness to CPAP therapy, and potential daytime fatigue could contribute to these findings. Accordingly, the literature on PVT performance following CPAP therapy offers mixed results. While some studies have demonstrated improvements in vigilance post-treatment, particularly in individuals with severe OSA and marked daytime sleepiness, others have reported no significant changes despite improvements in other cognitive domains, such as verbal memory or executive functions [7].

This discrepancy underscores the need for alternative or complementary measures of vigilance that might be more sensitive to subtle changes induced by CPAP therapy. For example, tasks that capture complex attentional processes or real-world vigilance scenarios could provide a more nuanced understanding of CPAP’s impact on cognitive functioning. Additionally, combining objective measures like the PVT with subjective assessments of vigilance might offer a more comprehensive view of treatment effects. These mixed findings on vigilance contrast with the improvements observed in other cognitive domains. For instance, in a different study, no significant changes in PVT performance were found with OSA treatment, whereas verbal memory showed improvement after the first night of treatment and remained stable up to three months later [7]. Our study found that phonological fluency remained unchanged across sessions, while semantic fluency improved significantly after 3 months of CPAP use and remained stable after 6 months. This suggests that CPAP therapy has a more pronounced effect on semantic fluency, likely due to enhancements in memory retrieval and executive functions [52]. The significant improvement in semantic fluency highlights the cognitive benefits of CPAP therapy in semantic processing [53]. These findings emphasize that different types of verbal fluency tasks may be influenced differently by treatments, underscoring the importance of evaluating specific cognitive domains when assessing verbal fluency [53]. These results add to the growing body of evidence supporting the cognitive benefits of CPAP treatment in individuals with OSA, shedding light on the nuanced effects of CPAP therapy on different aspects of cognitive performance [54]. However, the repeated administration of cognitive tasks across different time points raises the possibility of repetition effects that can impact observed results [55]. It is crucial to consider the influence of task familiarity, repetition, and practice effects when interpreting cognitive task performance to ensure accurate assessment and understanding of cognitive abilities and interventions. Although improvements in delayed recall scores and semantic fluency were observed after six months of CPAP therapy, these enhancements may partly stem from practice effects due to prior task exposure. While repetition effects could contribute to these enhancements, the consistency of these improvements with the existing literature on the cognitive benefits of CPAP therapy suggests that these effects are not solely due to repetition but are likely augmented by the treatment itself [43]. Studies have shown that cognitive functioning, including working memory, long-term verbal memory, and short-term visuospatial memory, can improve after CPAP therapy, indicating the positive impact of treatment on cognitive performance [53]. To address and consider repetition effects, it is crucial to acknowledge that some cognitive improvements may be influenced by participants’ increasing familiarity with the tasks. This familiarity could be particularly relevant for tasks requiring speed and accuracy, such as the Stroop task, where repeated exposure might lead to performance gains independent of cognitive recovery or enhancement [56]. Conversely, the lack of improvement in certain tasks like phonological fluency and short-term memory measures may suggest that these tasks are less susceptible to practice effects or that CPAP therapy selectively enhances specific cognitive domains over others [46]. While repetition effects from task familiarity and practice may influence cognitive performance to some extent, the overall cognitive improvements observed after CPAP therapy are likely a combination of these effects and the therapeutic benefits of the treatment. By considering the interplay between repetition effects and treatment outcomes, researchers and clinicians can better interpret cognitive changes in individuals undergoing CPAP therapy for obstructive sleep apnea.

This dichotomy in cognitive function improvements raises important questions about the role of cognitive fatigue, which seems to impact various cognitive domains differently. In this study, we used the TloadDback task to generate distinct and objective levels of cognitive fatigue (CF) while maintaining uniform task complexity. The TloadDback task requires participants to continuously engage in a working memory task that involves updating and manipulating information. To increase cognitive demands and induce CF, we regulated the processing time for ongoing data based on the capabilities of each participant. This control is crucial given the major inter-individual differences in net cognitive ability [57], which implies varied perceptions of task difficulty across different individuals. Our method sought to stabilize learning-related outcomes before objectively examining the effects of CF [18]. Moreover, the calibration time for adjusting the task to each participant’s capabilities decreased significantly over time, with gradually faster calibration after 3 months and even more after 6 months of treatment. This reduction in calibration time suggests improved processing efficiency resulting from the treatment. The shorter processing time indicates a potential alleviation of cognitive fatigue and an enhancement of cognitive function over the course of the treatment. Performance during TloadDback practice was segmented into four intervals to evaluate its progression over time in the face of a fatigue-inducing task. The CF-inducing task results indicated a significant improvement in performance within blocks during the final session, particularly between the first and third block. This improvement may be attributed to the effects of CPAP treatment on cognitive abilities [46,58,59], as evidenced by the enhanced interstimulus interval (ISI) during the task, making it more challenging compared to earlier sessions [49]. However, the performance in the last block did not continue increasing, as indicated by the absence of notable changes in the Delta scores for cognitive fatigue across sessions, even after 6 months of CPAP use. This suggests the anticipated impact of CF over extended periods [18]. It was expected that performance declines across blocks, due to the normal difficulty in maintaining proficient processing of incoming information, highlighting the distinct and substantial impact of the task in inducing CF [18]. This gradual decline illustrates a core aspect of the human processing system referred to as “the principle of graceful degradation”, where performance declines when at least two cognitive processes consume the same limited resources [60]. Thus, prolonged cognitive demands would lead to subjective CF and a continuous decline in performance over the 16 min task [18]. Additionally, the observed drop in motivation between pre- and post-task evaluations, coupled with the increase in subjectively evaluated CF over the same period, further supports the notion of cognitive fatigue affecting task performance [49]. This is consistent with Ackerman’s theory [61], which attributes performance decline to a loss of interest in a task characterized by time pressure and verbal content, particularly when the task continues without breaks. Furthermore, after 6 months of CPAP treatment, the post-task sleepiness did not increase, indicating that the subjects could clearly distinguish between CF and sleepiness. This differentiation underscores the specific effects of CPAP on sleepiness after 6 months of treatment [62] and highlights the distinct impact of the task in inducing CF [18]. Varandas and collaborators [63] further emphasize that CF tends to intensify more rapidly than sleepiness, with tasks of lower cognitive demand leading to a greater increase in sleepiness compared to CF. Here, participants performed at the peak of their capabilities, based on the calculation of their ISI. Overall, subjective evaluations of sleepiness, CF, and stress generally increased post-task in each session, except for sleepiness in the third session, as discussed above. This pattern likely reflects the cumulative cognitive load and fatigue associated with the TloadDback task, despite participants showing overall improvements in cognitive performance with CPAP therapy. The absence of significant differences in Delta scores for sleepiness, stress, and motivation across sessions suggests that while CPAP treatment enhances cognitive function, it does not substantially alter the subjective experience of fatigue, sleepiness, motivation, and stress over time. This outcome may be attributed to the task’s calibration, which was designed to engage and challenge each participant according to their specific cognitive abilities [18]. Consequently, the task consistently triggered cognitive effort and fatigue, irrespective of performance improvements.

CPAP has been shown to enhance adherence, improve health-related quality of life, reduce daytime sleepiness, and increase overall functioning in individuals with OSA [64]. Good adherence to CPAP therapy is defined as the use of CPAP treatment for at least 4 h per night in 70% of nights [65]. In our study, adaptation to CPAP therapy varied among participants, reflecting the diverse experiences and challenges associated with the initiation of this treatment. Half of the participants (11 out of 22) reported adapting within less than a week, indicating a relatively quick adjustment for many individuals. This rapid adaptation could be attributed to factors such as motivation, immediate relief from OSA symptoms, and effective initial support and education provided by healthcare professionals [66]. Three participants adapted within two weeks, while another three participants took less than a month to feel comfortable using CPAP. For two participants, the adaptation period extended to less than two months. Notably, three participants required more than six months to adapt, highlighting the need for continuous support and monitoring to address any issues that may arise, such as discomfort, claustrophobia, or equipment-related challenges [67]. Our study revealed significant correlations between CPAP adherence and specific cognitive performance outcomes. Higher CPAP adherence was associated with more stable cognitive performance across task blocks, as indicated by the negative correlation with Delta scores. Additionally, adherence was linked to improvements in verbal fluency, with significant positive correlations for both phonological and semantic fluency scores, reflecting enhanced lexical access and semantic processing. Furthermore, the observed negative correlation with Stroop denomination time suggests that greater adherence may facilitate more efficient automatic linguistic processes. These findings underscore the critical role of CPAP adherence in promoting cognitive recovery, and align with prior literature associating regular CPAP use, particularly exceeding 4 h per night, with enhancements in memory, attention, and executive functioning. They also resonate with the subjective improvements in quality of life reported in our study. The correlation analysis revealed several key insights into the factors influencing CPAP adherence and their relationships with patient experiences. While CPAP-related constraints and issues did not show a significant correlation with mean CPAP use (*p* > 0.05), the varied adaptation periods suggest these challenges may still affect comfort and long-term adherence. The absence of a significant correlation between CPAP-related constraints and mean usage hours suggests that patients may initially tolerate discomfort without it affecting usage, but persistent discomfort may reduce adherence over time [68]. The prolonged adaptation period is particularly relevant when considering the significant negative correlation found between mean CPAP use and variables in the usage habits and behaviors group. It suggests that inconsistent usage patterns, such as frequent mask abandonment, could be linked to initial difficulties in adapting to CPAP. Research indicates that discomfort with the CPAP mask is a significant predictor of adherence [68]. Participants who took longer to adapt may have developed usage habits that ultimately reduced their overall adherence, despite not directly reporting CPAP-related constraints as a primary issue [69]. Additionally, while we did not observe a significant correlation between CPAP use and self-reported improvements in quality of life, prolonged adaptation periods may have delayed the potential benefits, affecting perceptions of CPAP’s impact on quality of life [70]. Notably, a significant positive correlation was observed between sleep continuity and CPAP-related discomfort and constraints, suggesting that individuals with better sleep continuity may be more aware of or sensitive to the discomfort caused by the CPAP device, or that issues related to CPAP therapy could negatively impact sleep quality, leading to disrupted sleep continuity.

The subjective evaluation of the impact of CPAP on participants’ lives demonstrated a significant improvement from the beginning of the experiment to after three months of treatment. The mean scores indicated a consistent enhancement in the subjective experience of the participants, with Session 1 showing higher scores (indicating worse effects) compared to Session 2. These findings highlight the substantial positive impact of CPAP therapy on the quality of life of individuals with OSA, suggesting that CPAP benefits extend beyond clinical measures to significantly enhance subjective well-being and life satisfaction [71]. Common challenges faced by our CPAP users included difficulties in adjusting to a new sleep position due to the equipment, discomfort from the mask, issues with finding the right mask fit, leaks, noise from the machine, nasal congestion, and mouth dryness. These challenges can lead to a persistent subjective experience of discomfort and fatigue, impacting treatment compliance and efficacy [72]. Research indicates that approximately two-thirds of CPAP users experience mask discomfort, nasal/pharyngeal dryness, or pressure intolerance, which can contribute to non-adherence to therapy [73]. Moreover, only around 54.3% of participants adhere to CPAP treatment using standard protocols, highlighting the challenges in maintaining compliance with CPAP therapy [74]. Despite the discomfort associated with CPAP masks, patients who regularly use CPAP often find that the benefits of treatment outweigh the mask’s discomfort [75]. This gradual decrease in discomfort observed in this study likely results from participants’ growing familiarity with the equipment and improved tolerance. This could be due to initial support and education from healthcare professionals [76], as well as secondary benefits in subjective sleep parameters [77,78]. Measures related to mask usage consistency, such as the ability to keep the mask on during the night, continuous CPAP use throughout the night, and putting the mask back on after each awakening, did not show significant differences across sessions, indicating that once participants establish a routine with CPAP therapy, their usage patterns remain stable.

A significant improvement in mood across sessions was also reported. Previous studies have found that CPAP therapy positively impacts quality of life, mood, and daytime sleepiness [69,79]. This improvement in mood can be attributed to the alleviation of OSA symptoms, leading to enhanced sleep quality and reduced fatigue. These positive changes are known to have a significant impact on emotional well-being, ultimately enhancing overall quality of life and promoting adherence to CPAP therapy [80,81]. Studies have shown that CPAP therapy can lead to a reduction in depressive symptoms, anxiety, and sleepiness, contributing to an overall improvement in mood [81]. The positive effects on mood have been observed to persist over time, with some studies reporting sustained improvements up to 12 months [80]. Additionally, the relief from symptoms such as daytime sleepiness and improved functional status can further contribute to enhanced mood and emotional well-being [82].

There was also a trend towards significance for a decrease in sudden bouts of fatigue during the day, particularly after three months of CPAP use. Although not statistically significant, this trend suggests that prolonged CPAP therapy may help in reducing daytime fatigue, as a result of better sleep efficiency and fewer disruptions during the night [83]. Subjective evaluations of the occurrence such as migraines and shortness of breath did not show significant changes across sessions. This suggests that while CPAP therapy addresses primary symptoms of OSA, some related symptoms may persist and warrant further investigation and management [84].

After three months of CPAP therapy, significant improvements were observed in various aspects related to subjective sleep quality, including sleep latency, duration, disturbances, and overall satisfaction, as measured by the Pittsburgh Sleep Quality Index (PSQI) [28]. These improvements were maintained after six months of CPAP use. The St-Mary’s Sleep Questionnaire [33], which assesses the subjective sleep quality of the previous night, confirmed several improvements following CPAP therapy. After 3 months of CPAP use, sleep duration and depth showed significant improvement, reflecting a positive impact on sleep architecture and quality [85]. These improvements were maintained after 6 months, at which point sleep quality also significantly improved, suggesting sustained and progressive benefits of CPAP on overall sleep patterns. This improvement in sleep depth may be attributed to CPAP’s ability to enhance features of sleep architecture, such as slow oscillation and spindle electroencephalography (EEG) activity, which promote sleep depth and neuronal homeostasis [85]. Additionally, CPAP treatment was associated with a decrease in intercurrent awakenings related to CPAP use after 6 months of use, suggesting enhanced sleep continuity and reduced disruptions during the night [85]. This reduction in nighttime disruptions suggests that individuals gradually adapted to the CPAP device, leading to fewer awakenings and more consolidated sleep over time. Mental and physical fatigue, assessed by the Brugmann Fatigue Scale (BFS) [30], showed improvement following three months of CPAP therapy, and these improvements were maintained after six months of daily use. Similarly, research has indicated that CPAP treatment can lead to a decrease in symptoms of anxiety and depression in patients with OSA, with statistically significant reductions observed after several years of treatment compared to usual care alone [86]. Specifically, anxiety levels decreased significantly after three months of CPAP use and were maintained after six months of continuous treatment. This is supported by studies showing a reduction in anxiety symptoms from baseline to follow-up during CPAP therapy [87]. In terms of depression, symptoms decreased from mild depression in Session 1 to absence of depression after three months of CPAP use, although no further improvements were observed after six months. This stability could be attributed to the complex interplay between CPAP therapy and depressive symptoms. While some studies have reported a small improvement in depressive symptoms with CPAP treatment compared to control groups [88], other research has highlighted the need for careful screening of patients with OSA for depressive disorders to ensure comprehensive management [89].

Although our study demonstrates significant improvements in cognitive functioning and quality of life following CPAP therapy, it is important to note that the extent of cognitive recovery may vary among individuals. Given the variability in treatment response, a one-size-fits-all approach is insufficient for maximizing the benefits of CPAP therapy. Personalized strategies, such as adjusting CPAP pressure settings, providing enhanced support for adherence, and addressing specific comorbidities, are crucial for optimizing outcomes. For instance, patients with more severe baseline impairments might benefit from cognitive rehabilitation in parallel with CPAP therapy, while those facing mask discomfort may require more tailored device adjustments and support. Tailoring treatment to individual needs not only enhances adherence but also ensures that therapeutic goals are met, especially in domains like cognitive recovery and quality of life.

## 5. Limitations

While this study offers insights into the benefits of CPAP therapy for cognitive and subjective outcomes, a few limitations warrant consideration. The relatively small sample size may limit the broader generalizability of the findings, as well as the depth of statistical analyses. However, a power analysis confirmed that the equivalent sample size was sufficient to detect medium effect sizes with adequate statistical power (see Section 3.4). Additionally, although our study effectively demonstrates improvements over a three- to six-month period, the long-term sustainability of these benefits remains an area for further exploration. The absence of a control group, while a constraint, highlights an opportunity for future research to more precisely isolate the effects of CPAP therapy from other potential influencing factors. While baseline cognitive performance was assessed at the start of CPAP therapy, premorbid cognitive functioning remains unknown. Educational attainment, ranging from secondary to university levels, serves as an indirect indicator of premorbid cognitive potential and highlights the diversity within the sample. This variability may contribute to differences in cognitive outcomes post-treatment. Variability in CPAP adherence among participants could influence the degree of observed improvement in subjective well-being. The correlation analysis revealed that while usage habits and behaviors showed a significant negative correlation with adherence, factors such as CPAP-related constraints and issues, as well as quality of life improvements, did not significantly correlate with adherence. This suggests that different aspects of adherence might influence the results in varied ways. Moreover, the reliance on self-reported measures for subjective well-being introduces potential biases and variability. Future research could benefit from larger sample sizes, and from incorporating additional objective assessments of CPAP adherence, such as physiological indicators. Furthermore, while the selected cognitive tasks provided valuable insights, exploring a broader range of executive function tasks in subsequent studies could help to further elucidate the potential impact of CPAP therapy in these domains. Finally, exploring a wider range of tasks and real-world scenarios in subsequent research could enhance our understanding of the generalizability of these findings. Despite these limitations, this study significantly advances our knowledge of CPAP therapy’s impact and sets a solid foundation for future investigations into its long-term benefits and broader applications.

## 6. Conclusions

This study aimed to explore the cognitive and subjective effects of continuous positive airway pressure (CPAP) therapy over a three- to six-month period in individuals with obstructive sleep apnea (OSA). Our results indicate that CPAP therapy significantly improves subjective sleep quality, and cognitive functions including episodic memory, working memory, verbal fluency, and inhibition, over time. Performance improvements were observed in a task inducing cognitive fatigue, with notable enhancements after six months of CPAP use. These findings suggest that regular CPAP use not only mitigates the immediate physiological disturbances caused by OSA but also facilitates cognitive recovery and daily functioning. Participants also reported substantial gains in subjective well-being, including increased quality of life, reduced daytime sleepiness, and improved mood. These improvements highlight the broader psychosocial benefits of CPAP therapy and emphasize the importance of integrating patient-reported outcomes into the management of OSA.

## Figures and Tables

**Table 1:** Results for the pre-testing questionnaires across sessions.

						95% CI		Post-Hocs		
Measure	LMM		Session	Estimate	SE	Lower	Upper	Comparison	t	*p*
	F	*p*								
PSQI	8.49	0.001 ***	1	8.27	0.64	7.01	9.53	S1–S2	3.42	0.005 **
			2	5.67	0.64	4.41	6.93	S1–S3	3.18	0.007 **
			3	5.28	0.68	3.96	6.61	S2–S3	0.59	0.57
BFS (Mental)	15.14	<0.001 ***	1	5.67	0.65	4.39	6.96	S1–S2	4.76	<0.001 ***
			2	3.48	0.66	2.20	4.77	S1–S3	4.75	<0.001 ***
			3	3.04	0.68	1.71	4.37	S2–S3	0.67	0.51
BFS (Physical)	6.10	0.007 **	1	5.10	0.59	3.95	6.25	S1–S2	2.70	0.02 *
			2	3.66	0.59	2.51	4.81	S1–S3	2.76	0.02 *
			3	3.53	0.62	2.31	4.75	S2–S3	0.19	0.86
FSS	6.51	0.004 **	1	4.74	0.36	4.04	5.44	S1–S2	1.98	0.07
			2	3.94	0.36	3.23	4.65	S1–S3	3.16	0.007 **
			3	3.44	0.38	2.71	4.18	S2–S3	1.32	0.37
CHQ-Behavioral rhythms	2.25	0.12	1	20.50	1.92	16.73	24.27	S1–S2	0.65	0.53
			2	18.13	1.92	14.36	21.90	S1–S3	2.24	0.09
			3	15.57	1.99	12.66	20.47	S2–S3	1.23	0.24
CHQ-Distinctness	11.48	<0.001 ***	1	22.30	1.28	19.79	24.81	S1–S2	3.35	0.004 **
			2	17.81	1.28	15.31	20.32	S1–S3	3.06	0.009 **
			3	17.15	1.32	14.56	19.74	S2–S3	0.43	0.67
HADRS-Depression	3.78	0.03 *	1	7.63	1.16	5.35	9.90	S1–S2	2.70	0.02 *
			2	4.30	1.19	1.97	6.64	S1–S3	1.66	0.12
			3	4.18	1.22	1.78	6.57	S2–S3	0.00	1.00
HADRS-Anxiety	9.71	<0.001 ***	1	11.76	1.43	8.96	14.56	S1–S2	2.06	0.05 *
			2	7.27	1.46	4.41	10.14	S1–S3	3.53	0.004 **
			3	4.36	1.50	1.43	7.93	S2–S3	1.63	0.13

Note. PSQI = Pittsburgh Sleep Quality Index; BFS = Brugmann Fatigue Scale; FSS = Fatigue Severity Scale; CHQ = Chronotype Questionnaire; HADRS = Hospital Anxiety and Depression Scale. S1 = Session 1 (start of the experiment), S2 = Session 2 (after three months of CPAP treatment), S3 = Session 3 (after six months of CPAP treatment). * *p* < 0.05, ** *p* < 0.01, *** *p* < 0.001).

**Table 2:** Outcomes of the CPAP use questionnaire.

	Session 1		Session 2		Session 3		Statistics
	Mean	95%CI [Lower; Upper]	Mean	95% CI [Lower; Upper]	Mean	95% CI [Lower; Upper]	
Practical constraints	0.20	0.03; 0.72	0.04	0.002; 0.50	0.02	5.38 × 10^−4^; 0.53	$\chi^{2}$ = 5.09, *p* = 0.08
Mask discomfort	0.92	0.67; 0.99	0.53	0.29; 0.76	0.42	0.18; 0.70	$\chi^{2}$ = 11.69, *p* = 0.003
Spouse inconvenience	1.09 × 10^−4^	2.66 × 10^−7^; 0.04	5.34 × 10^−7^	2.22 × 10^−16^; 0.86	5.93 × 10^−5^	8.52 × 10^−8^0.04	$\chi^{2}$ = 3.50, *p* = 0.17
Keeping the mask	0.39	0.09; 0.80	0.21	0.04; 0.65	0.25	0.04; 0.73	$\chi^{2}$ = 0.93, *p* = 0.63
CPAP continuous use	0.40	0.20; 0.64	0.47	0.26; 0.70	0.50	0.25; 0.75	$\chi^{2}$ = 0.40, *p* = 0.82
Mask back	0.66	0.34; 0.88	0.41	0.17; 0.70	0.60	0.27; 0.86	$\chi^{2}$ = 1.86, *p* = 0.40
Occasional abandonment	0.31	0.12; 0.60	0.24	0.08; 0.52	0.15	0.03; 0.46	$\chi^{2}$ = 1.25, *p* = 0.54
Frequent abandonment	0.10	0.02; 0.35	0.24	0.09; 0.49	0.31	0.13; 0.58	$\chi^{2}$ = 2.74, *p* = 0.25
Full night with the mask	0.003	0.003; 0.003	6.23 × 10^−4^	6.19 × 10^−4^; 6.27 × 10^−4^	3.80 × 10^−4^	3.77 × 10^−4^; 3.82 × 10^−4^	$\chi^{2}$ = 1.49, *p* = 0.48
Bouts of fatigue	0.67	0.33; 0.89	0.92	0.61; 0.99	0.61	0.26; 0.88	$\chi^{2}$ = 5.47, *p* = 0.06
Mood	0.22	0.08; 0.50	0.73	0.45; 0.90	0.63	0.34; 0.84	$\chi^{2}$ = 10.02, *p*= 0.007
Concentration	0.19	0.05; 0.51	0.43	0.17; 0.74	0.34	0.10; 0.71	$\chi^{2}$ = 2.25, *p* = 0.32
Migraines	0.49	0.05; 0.95	0.59	0.07; 0.96	0.91	0.16; 1.00	$\chi^{2}$ = 2.61, *p* = 0.27

Note: All variables are binary. Mask back = Putting the mask back on after each awakening; Occasional abandonment = Occasional abandonment of the mask during the night; Frequent abandonment = Frequent abandonment of the mask during the night.

**Table 3:** Correlations between average CPAP use and key variables.

Variables		Pearson’s r	*p*-Value
Average CPAP Utilization (hours)	CPAP Treatment Follow-up and Improvements in Quality of Life	0.01	0.92
Average CPAP Utilization (hours)	Usage Patterns and Adherence Issues	−0.52	<0.001 ***
Average CPAP Utilization (hours)	CPAP-Related Discomfort and Constraints	−0.01	0.92
Average CPAP Utilization (hours)	Sleep Continuity	−0.09	0.50
Sleep Continuity	CPAP-Related Discomfort and Constraints	0.33	0.02 *

Note. * *p* < 0.05, *** *p* < 0.001.

**Table 4:** Outcomes of the neuropsychological battery in each of the three sessions.

	Session 1	Session 2	Session 3	Statistics
RLS 15-RM	12.38 (5.70)	12.98 (0.92)	13.66 (0.53)	F(2,39.07) = 0.67, *p* = 0.52
RLS 15-%RLTC	73.66 (23.06)	98.11 (38.74)	90.56 (9.19)	F(2,36.01) = 6.29, *p* = 0.005 **
RLS 15-RD	13.08 (2.57)	14.39 (1.20)	14.93 (0.27)	F(2,31.15) = 7.09, *p* = 0.003 **
Stroop-DenoTI	31.29 (6.00)	29.28 (5.18)	30.00 (7.05)	F(2,32.33) = 6.51, *p* = 0.004 **
Stroop-InterTI	55.82 (15.55)	46.44 (9.82)	46.57 (12.85)	F(2,33.68) = 13.97, *p* < 0.001 ***
Stroop-InterTT	110.39 (28.53)	96.50 (18.55)	98.29 (27.63)	F(2,34.63) = 6.55, *p* = 0.004 **
Stroop-Inter-DenoT	45.96 (21.55)	33.44 (11.92)	35.92 (20.07)	F(2,35.83) = 3.25, *p* = 0.05 *
Digit span (in order)	5.75 (1.03)	6.32 (1.16)	6.43 (1.16)	F(2,35.85) = 3.35, *p* = 0.05 *
Digit span (in reverse)	4.42 (1.28)	5.16 (1.34)	4.79 (1.31)	F(2,36.27) = 7.92, *p* = 0.002 **
Phonological fluency	21.83 (7.99)	23.00 (7.86)	25.36 (9.31)	F(2,35.39) = 2.00, *p* = 0.15
Semantic fluency	28.79 (8.99)	32.53 (7.98)	34.36 (9.72)	F(2,36.23) = 7.41, *p* = 0.002 **

Note: RLS-15 = episodic memory task; RM = mean of the immediate recall trials, % RLTC = percentage of words recalled in at least two consecutive immediate recall trials from the total number of words recalled, RD = delayed recall; DenoTI = intermediate time in the denomination subtask of the Stroop task; InterTI = intermediate time in the interference subtask of the Stroop task; InterTT = total time in the interference subtask of the Stroop task; Inter-Deno T = Inhibition score, measured as the difference in response speed between denomination and interference conditions of the Stroop task; Digit span (in order) = subtest of the WAIS. Digit span (in reverse) = subtest of the WAIS. PVT = Psychomotor Vigilance Task; min = minimal score. Data are mean (±SD) scores, * *p* < 0.05, ** *p* < 0.01, *** *p* < 0.001.

**Table 5:** Summary of significant correlations: CPAP use and cognitive performance.

Variable	Correlation Coefficient (r)	*p*-Value	Interpretation
Delta Scores	r= −0.268	*p* = 0.044	Suggests more stable cognitive performance across task blocks with higher CPAP adherence.
Phonological Fluency	r = 0.331	*p* = 0.012	Greater CPAP use associated with improved lexical access and word generation.
Semantic Fluency	r = 0.385	*p* = 0.003	CPAP adherence linked to enhanced semantic processing.
Stroop Denomination Time (Deno_Tl)	r = −0.271	*p* = 0.049	Higher CPAP use facilitating the automatic processing required for rapid word naming.

Note: Delta Scores: Represents the variation in performance across the first and last blocks of the TloadDback task (cognitive fatigue). A negative correlation indicates more stable cognitive performance with higher CPAP adherence. Phonological Fluency: Assesses the ability to generate words starting with a specific letter within a limited time. Positive correlation reflects improved lexical access. Semantic Fluency: Measures the ability to generate words within a semantic category (e.g., animals). Positive correlation suggests enhanced semantic processing. Stroop Denomination Time (Deno_Tl): Indicates the time taken to name colors in a word-reading task. Negative correlation reflects greater efficiency in automatic linguistic processes.

**Table 6:** Outcomes of the VAS in each of the three sessions.

	Session 1		Session 2		Session 3	
	Pre-Task	Post-Task	Pre-Task	Post-Task	Pre-Task	Post-Task
Sleepiness	2.37 (2.20)	3.75 (3.10)	1.75 (1.99)	2.99 (2.63)	2.10 (2.21)	2.73 (2.67)
Cognitive Fatigue	3.95 (2.34)	5.33 (2.79)	2.69 (2.57)	3.98 (2.56)	2.83 (2.42)	4.24 (2.48)
Stress	2.49 (2.11)	4.71 (2.30)	1.45 (1.94)	3.14 (2.29)	1.11 (1.31)	3.43 (2.57)
Motivation	6.83 (2.15)	5.66 (2.20)	6.72 (2.62)	5.36 (2.12)	7.04 (2.36)	5.13 (2.61)

Note: Data are mean (±SD) scores. Session 1: start of the experiment, Session 2: after three months of CPAP treatment, Session 3: after six months of CPAP treatment.

**Table 7:** Results of the exploratory analysis for the variables from the CPAP feedback questionnaire.

Factor	Eigenvalue	Description	Variables	Explained Variance
Factor 1: CPAP Treatment Follow-up and Quality of Life Improvements	3.24	Reflects the positive impact of CPAP on daily life and well-being.	- Impact of diagnosis and treatment on daily life	19.05%
			- Mood improvement	
			- Reduced daytime fatigue	
			- Enhanced concentration and memory	
			- Reduction in headaches	
			- Mask-related discomfort	
Factor 2: Usage Patterns and Adherence Issues	2.79	Represents challenges with adherence and usage patterns.	- Never using the mask for a full night	16.39%
			- Frequent abandonment of the mask	
			- Difficulty maintaining the mask	
			- Continuous use of CPAP most nights	
			- Reduction in headaches	13.76%
Factor 3: CPAP-Related Discomfort and Constraints	2.34	Captures discomfort and constraints related to CPAP.	- Discomfort for the partner	
			- Practical constraints of CPAP	
			- Occasional abandonment of the mask	
			- Perception that the machine prevents sleep	
Factor 4: Sleep Continuity	1.64	Indicates improved sleep continuity with CPAP.	- Continuous CPAP use most nights	9.66%
			- Occasional abandonment of the mask	
			- Fewer nighttime awakenings to urinate	
			- Reapplying the mask after each awakening	

## Data Availability

The data are accessible from the corresponding author upon reasonable request.

## Supplementary Materials

The following supporting information can be downloaded at: https://www.mdpi.com/article/10.3390/clockssleep6040051/s1, Table S1: Outcomes of the St-Mary’s Sleep Questionnaire during the first day of testing; Table S2: Outcomes of the St-Mary’s Sleep Questionnaire during the second day of testing; Table S3: Detailed outcomes on the Stroop task; Table S4: Detailed outcomes on the Psychomotor Vigilance task.
