# Supplementary material for: Impact of CPAP Therapy on Cognition and Fatigue in Patients with Moderate to Severe Sleep Apnea: A Longitudinal Observational Study
Source: Clocks Sleep. 2024 Dec 23;6(4):789–816. doi: 10.3390/clockssleep6040051 (PMC11674386; doi:10.3390/clockssleep6040051)
Supplement: Supplementary file 1 [file clockssleep-06-00051-s001.zip › clockssleep-3317691-supplementary.pdf]

Table S1: Outcomes of the St-Mary's Sleep Questionnaire during the first day of testing

	Session 1	Session 2	Session 3	Statistics
Preparation to sleep (time)	23.18 (1.33)	22.96 (1.23)	22.77 (1.25)	F(1,18.21) = 0.46, p = 0.50
Sleep (time)	23.97 (1.41)	23.71 (1.20)	23.40 (1.23)	F(1,18.91) = 1.57, p = 0.23
Wake (time)	8.04 (3.77)	7.45 (1.16)	6.98 (1.45)	F(1,18.14) = 1.03, p = 0.32
Out of bed (time)	7.76 (1.37)	7.94 (1.17)	7.34 (1.47)	F(1,18.14) = 1.03, p = 0.32
Sleep Difficulty	1.57 (1.12)	1.14 (0.48)	1.31 (0.48)	F(1,22.70) = 1.31, p = 0.27
Sleep Latency (min)	12.62 (9.37)	12.10 (9.17)	14.69 (12.55)	F(1,20.01) = 0.26, p = 0.62
Sleep Quality	4.10 (1.22)	4.43 (1.21)	4.81 (0.91)	F(1,36.72) = 6.72, p = 0.01**
Sleep Depth	4.76 (1.67)	5.52 (1.78)	5.50 (1.63)	F(1,17.89) = 5.79, p = 0.03 *
Sleep Satisfaction	3.38 (1.07)	3.62 (1.24)	3.44 (1.32)	F(1,19.68) = 0.30, p = 0.59
Intercurrents awakenings (nb)	2.43 (1.86)	1.50 (1.19)	1.75 (1.34)	F(1,20.57) = 3.32, p = 0.08
CPAP Intercurrents awakenings (nb)	1.57 (1.89)	1.24 (1.20)	0.86 (1.17)	F(1,21.19) = 4.65, p = 0.04 *
Night Sleep duration (h)	6.71 (1.54)	7.59 (1.30)	7.34 (1.07)	F(1,35.70) = 5.51, p = 0.03 *
CPAP usage duration (h)	9.87 (15.90)	7.84 (5.64)	6.38 (1.59)	F(1,24.34) = 0.73, p = 0.40
Nap duration (min)	6.95 (20.35)	13.80 (0.00)	12.00 (27.31)	F(1,16.05) = 0.003, p = 0.96
Sleepiness at wake	3.62 (1.24)	4.48 (0.93)	4.00 (0.89)	F(1,37.30) = 2.45, p = 0.13

Note: Data are mean (± SD) scores. Sleep Difficulty = Difficulty falling asleep, 1 = none or very little, 2 = some difficulty, 3 = a lot of difficulty, 4 = an extreme difficulty. Sleep Latency (min) = scores in minutes. For Sleep Quality scores, 1 = Very bad, 2 = bad, 3 = bad enough, 4 = good enough, 5 = good, 6 = very good. For Sleep depth scores, 1 = very light, 2 = light, 3 = quite light, 4 = moderately light, 5 = moderately deep, 6 = quite deep, 7 = deep, 8 = very deep. For Sleep Satisfaction, 1 = very unsatisfied, 2 = moderately satisfied, 3 = slightly satisfied, 4 = satisfied enough, 5 = very satisfied. Intercurrents awakenings (nb) = scores in numbers of awakenings within the night. CPAP Intercurrents awakenings (nb) = scores in numbers of awakenings within the night due to the use of the CPAP treatment. Night Sleep duration (h) = scores in hours. Nap duration = scores in minutes. For Sleepiness scores, 1 = very sleepy, 2 = quite sleepy, 3 = a little sleepy, 4 = lucid, 5 = alert, 6 = very alert. * $p < 0.05$, ** $p < 0.01$.

Table S2: Outcomes of the St-Mary's Sleep Questionnaire during the second day of testing

	Session 1	Session 2	Session 3	Statistics
Preparation to sleep (time)	22.52 (3.38)	23.31 (1.24)	22.94 (0.90)	F(2,38.16) = 0.76, p = 0.48
Sleep (time)	23.63 (1.17)	23.77 (1.11)	23.55 (0.86)	F(2,34.44) = 0.21, p = 0.83
Wake (time)	7.27 (1.97)	7.50 (1.02)	6.41 (2.06)	F(2, 37.95) = 2.56, p = 0.09
Out of bed (time)	7.54 (1.93)	7.94 (1.05)	7.60 (0.93)	F(2, 37.38) = 1.02, p = 0.37
Sleep Difficulty	1.36 (0.73)	1.24 (0.63)	1.19 (0.54)	F(2, 39.45) = 0.53, p = 0.60
Sleep Latency (min)	23.86 (20.42)	18.33 (17.96)	14.94 (14.74)	F(2, 37.82) = 2.02, p = 0.15
Sleep Quality	4.14 (1.21)	4.48 (1.03)	4.31 (1.25)	F(2,37.66) = 0.63, p = 0.54
Sleep Depth	5.14 (1.42)	5.52 (1.60)	5.44 (1.59)	F(2,36.08) = 1.03, p = 0.37
Sleep Satisfaction	4.24 (1.26)	4.05 (1.47)	3.31 (1.49)	F(2,37.33) = 2.19, p = 0.13
Intercurrents awakenings (nb)	2.62 (1.80)	1.40 (1.50)	1.88 (2.00)	F(2,54) = 2.50, p = 0.09
CPAP Intercurrents awakenings (nb)	1.75 (2.07)	0.88 (1.27)	1.23 (2.24)	F(2,34.90) = 1.24, p = 0.30
Night Sleep duration (h)	6.59 (1.58)	7.41 (1.03)	6.48 (2.02)	F(2,35.94) = 1.18, p = 0.32
CPAP usage duration (h)	6.06 (1.91)	6.42 (2.24)	5.56 (1.97)	F(2,35.94) = 1.18, p = 0.32
Sleepiness at wake	3.55 (1.14)	3.81 (1.08)	3.94 (1.24)	F(2,37.38) = 1.02, p = 0.37

Note: Data are mean (± SD) scores. Sleep Difficulty = Difficulty falling asleep, 1 = none or very little, 2 = some difficulty, 3 = a lot of difficulty, 4 = an extreme difficulty. Sleep Latency (min) = scores in minutes. For Sleep Quality scores, 1 = Very bad, 2 = bad, 3 = bad enough, 4

= good enough, 5 = good, 6 = very good. For Sleep depth scores, 1 = very light, 2 = light, 3 = quite light, 4 = moderately light, 5 = moderately deep, 6 = quite deep, 7 = deep, 8 = very deep. For Sleep Satisfaction, 1 = very unsatisfied, 2 = moderately satisfied, 3 = slightly satisfied, 4 = satisfied enough, 5 = very satisfied. Intercurrents awakenings (nb) = scores in numbers of awakenings within the night. CPAP Intercurrents awakenings (nb) = scores in numbers of awakenings within the night due to the use of the CPAP treatment. Night Sleep duration (h) = scores in hours. For Sleepiness scores, 1 = very sleepy, 2 = quite sleepy, 3 = a little sleepy, 4 = lucid, 5 = alert, 6 = very alert.

Table S3: Detailed outcomes on the Stroop task.

	Session 1	Session 2	Session 3	Statistics
Stroop - DenoTT	66 (12.23)	66.75 (15.50)	63.83 (10.93)	F(2,34.28) = 2.36, p = 0.32
Stroop - DenoEC	1.33 (3.11)	0.75 (0.75)	0.50 (0.67)	F(2,36.83) = 0.51, p = 0.70
Stroop - DenoENC	0.08 (0.29)	0.00 (0.00)	0.00 (0.00)	F(2,54) = 1.80, p = 0.18
Stroop - LectTI	20.55 (2.07)	20.46 (3.75)	20.09 (0.67)	F(2,32.02) = 0.20, p = 0.82
Stroop - LectTT	45.50 (8.43)	44.00 (8.10)	44.25 (7.94)	F(2,34.10) = 1.74, p = 0.19
Stroop - LectEC	0.33 (0.89)	0.17 (0.39)	0.17 (0.39)	F(2,36.33) = 0.23, p = 0.80
Stroop - InterEC	2.08 (3.00)	1.67 (3.39)	2.17 (3.51)	F(2,34.80) = 0.79, p = 0.46
Stroop - InterENC	0.50 (1.45)	0.17 (0.39)	0.08 (0.29)	F(2,38.42) = 2.11, p = 0.14

Note: Data are mean (± SD) scores. DenoTT = Total time in the denomination subtask, DenoEC = Number of corrected errors in the denomination subtask, DenoENC = Number of uncorrected errros in the denomination subtask, LectTI = Intermediate time in the reading subtask, LectTT = Total time in the reading subtask, LectEC = Number of corrected errors in the reading subtask, LectENC = Number of uncorrected errors in the reading subtask, InterEC = Number of corrected errors in the interference subtask, InterENC = Number of uncorrected errors in the interference subtask.

Table S4: Detailed outcomes on the Psychomotor Vigilance task.

	Session 1	Session 2	Session 3	Statistics
Median	339.25 (64.40)	346.42 (37.42)	357.83 (81.99)	F(2,35.29) = 0.09, p = 0.91
Percentile 10	278.08 (39.30)	295.25 (26.47)	282.75 (32.96)	F(2,33.38) = 0.80, p = 0.46
Percentile 90	449.08 (100.91)	440.67 (62.86)	441.58 (90.80)	F(2, 33.38) = 0.80, p = 0.46
Mean	352.33 (65.76)	366.75 (41.45)	368.92 (71.03)	F(2,35.10) = 0.01, p = 0.99
SD	71.33 (27.68)	111.83 (151.37)	114.75 (149.37)	F(2,36.48) = 0.99, p = 0.38
Lapses > 500 ms	6.50 (10.85)	4.42 (4.87)	7.75 (15.50)	F(2,37.90) = 0.48, p = 0.62
Lapses > 2 SD	4.08 (1.38)	3.25 (1.42)	2.17 (1.12)	F(2,35.78) = 1.64, p = 0.21
Lapses < 2 SD	0.08 (0.29)	0.08 (0.29)	0.33 (1.16)	F(2,38.84) = 1.29, p = 0.29
Minimum (ms)	237.86 (29.22)	263.79 (28.64)	253.63 (30.05)	F(2,35.15) = 0.76, p = 0.48
Maximum (ms)	601.19 (188.53)	1062.61 (1477.18)	1081.28 (1436.18)	F(2,36.22) = 1.33, p = 0.28
Max - Min	363.34 (172.82)	798.82 (1481.38)	827.66 (1435.39)	F(2,36.25) = 1.27, p = 0.30
RRT	3.01 (0.45)	2.88 (0.31)	2.93 (0.50)	F(2,34.09) = 0.22, p = 0.80

Note: Data are mean (± SD) scores. SD = Standard Deviation; Minimum = Minimal Reaction Time; Maximum : Maximal Reaction Time; Max – Min = Maximal Reaction Time - Minimal Reaction Time ; RRT = Reciprocal Reaction Time.
